# Synthesis of Single Crystal 2D Cu_2_FeSnS_4_ Nanosheets with High-Energy Facets (111) as a Pt-Free Counter Electrode for Dye-Sensitized Solar Cells

**DOI:** 10.3390/ma16134743

**Published:** 2023-06-30

**Authors:** Jianming Wen, Suqin Chen, You Xu, Tuxiang Guan, Xiaoyan Zhang, Ningzhong Bao

**Affiliations:** State Key Laboratory of Materials-Oriented Chemical Engineering, College of Chemical Engineering, Nanjing Tech University, Nanjing 210009, China202161104120@njtech.edu.cn (S.C.);

**Keywords:** Cu_2_FeSnS_4_ nanosheets, high-energy facets, counter electrode, dye-sensitized solar cells

## Abstract

Two-dimensional Cu_2_FeSnS_4_ (CFTS) nanosheets with exposed high-energy facets (111) have been synthesized by a facile, scalable, and cost-effective one-pot heating process. The CFTS phase formation is confirmed by both X-ray diffraction and Raman spectroscopy. The formation mechanism of exposed high-energy facet CFTS growth is proposed and its electrochemical and photoelectrochemical properties are investigated in detail to reveal the origin of the anisotropic effect of the high-energy facets. Dye-sensitized solar cells (DSSC) achieve a favorable power conversion efficiency of 5.92% when employing CFTS thin film as a counter electrode, suggesting its potential as a cost-effective substitute for Pt in DSSCs.

## 1. Introduction

The efficiency of energy conversion devices relies heavily on the careful selection of electrode materials [1,2,3,4,5]. One such material that has been commonly used is Pt-based catalysts, known for their effectiveness in tri-iodide and oxygen reduction processes. However, these catalysts come with a drawback of high costs and stability issues, making them less desirable for the large-scale production of photovoltaic devices [6,7,8,9,10,11]. In light of these challenges, researchers have turned their attention toward 2D chalcogenides as promising alternatives due to their unique properties and environmental friendliness [6,7,8,9,10,11,12,13,14]. Particularly, with special exposed high-energy facets, 2D chalcogenide can promote charge migration across both the electrolyte and the photoelectrode interface and increase photon–matter interaction via multiple reflection and scattering at the electrolyte inter-face, which will provide much higher catalytic activity [15,16]. Hence, 2D chalcogenides with exposed high-energy facets are increasingly being considered the most favorable replacement for Pt toward counter electrodes in dye-sensitized solar cells (DSSCs) [17]. Nevertheless, the frequent inability to regulate the growth of exposed high-energy facets often results in their swift disappearance during crystal formation or utilization in catalytic processes. This phenomenon can be attributed to the significantly faster crystal growth rate perpendicular to a high-energy facet compared to the growth rate along the normal direction [18,19]. As a result, the synthesis of 2D chalcogenide with desired high-energy facets is still a challenge.

The quaternary semiconductor Cu_2_XSnS_4_ (X = Zn, Mn, Fe, Co, and Ni) has been widely investigated as a functional material for photovoltaic applications with optimal band gap, high absorption coefficient, and the presence of abundant earth elements [20,21,22]. Among these, as a naturally occurring mineral, low-cost and non-toxic Cu_2_FeSnS_4_ (CFTS) with band gaps of 1.3−1.5 eV has been shown to demonstrate promising applications in photoelectrochemical cells. Compared to Pt, the elements composing CFTS not only have the characteristics of abundant crystal content and low price but also possess environmental friendliness and low toxicity [23,24]. In this regard, CFTS has been considered a cheaper alternative to Pt for the development of Pt-free, non-toxic, and cost-effective counter electrodes in DSSCs [25,26]. Moreover, CFTS not only has the potential to replace Pt in the field of solar cells but also demonstrates impressive performance in areas such as photocatalytic hydrogen production. Therefore, the development of high-performance CFTS holds significant importance for the sustainable development of global energy [27].

To date, many reports have focused on synthesizing CFTS nanomaterials with various morphologies and improving their photoelectrochemical performance. Most of these investigations have resulted in CFTS nanorod, nanowire, nanoplate, and irregular nano-particle [25,26,28,29,30]. In our earlier work, we presented findings on the colloidal syntheses of various chalcogenide nanocrystals, including PbS, FeS_2_, CuCr_2_Se_4_, CuInS_2_, Cu_2_ZnSnS_4_, Cu_2_FeSnS_4_, and Culn_x_Ga_1−x_S(Se)_2_. These nanocrystals were synthesized with diverse shapes and sizes [31,32,33,34]. The synthesis processes for these materials entail the thermal decomposition and reaction of appropriate metal and chalcogenide precursors using either the hot-injection method or the solvothermal approach. However, to engineer chalcogenide with a 2D nanostructure and exposed high-energy facets remains largely unexplored. As our previous report indicated, high-energy crystal facets may possess higher adsorption energy toward I_3_^−^, thereby facilitating I_3_^−^ ion reduction and improving power conversion efficiency [35]. Moreover, 2D materials possess unique properties, such as a large aspect ratio and atomic-level thickness, which result in abundant surface-active atoms and edge-active sites [36,37]. These features make them highly attractive for catalytic processes [38,39]. The growth rate of high-energy crystal facets is usually faster than that of low-energy crystal facets, leading to the tendency of high-energy facets to disappear during the growth process [18,40]. In this regard, achieving a controllable preparation of 2D materials and directionally controlling their exposed crystal planes is an urgent problem to be solved.

Herein, we report the fabrication of 2D CFTS nanosheets with exposed high-energy facets (111) as effective electrode materials for DSSCs by a facile, scalable, and cost-effective one-pot heating process. CFTS thin films are deposited on FTO substrates by a simple spray-coating followed by annealing. To further research the intrinsic driving force for its anisotropic growth and energy barrier upon high-energy facet migration, the formation mechanism of exposed high-energy facet 2D growth is proposed. The electrochemical and photoelectrochemical properties of synthesized CFTS nanosheets are investigated in detail to reveal the origin of the anisotropic effect of the high-energy facets. Finally, our exposed high-energy facet CFTS nanosheets can be applied to a wide range of magnetic, electronic, and solar energy conversion devices [35].

## 2. Materials and Methods

### 2.1. Chemicals

The chemicals employed in the experiment were as follows, with their respective sources and purities: copper(II) acetylacetonate (Cu(acac)_2_, Aladdin, Shanghai, China, 97%), ferric chloride hexahydrate (FeCl_3_·6H_2_O, Aladdin, 99%), Tin(IV) chloride pentahydrate (SnCl_4_·5H_2_O, 99%), 1-dodecanethiol (1-DDT, Aladdin, 98%), formamide (CH_3_NO, Lingfeng Chemical Reagent, Shanghai, China, 99%), and ammonium sulfide ((NH_4_)_2_S, Tongya Chemical, Shanghai, China, S > 8%). The electrolyte (OPV-AN-1) used for DSSC containing lithium iodide (LiI), Iodine (I_2_), 4-tert-Butylpyridine (TBP), 1-Methyl-3-propylimidazolim iodide (PMII), and acetonitrile was purchased from OPV Tech Co., Ltd., Shenzhen, China The TiO_2_ photoanodes (thickness: ~600 nm, active area: ~0.16 cm^2^) and Di-tetrabutylammonium cis-bis(isothiocyanato)bis(2,2′-bipyridyl-4,4′-dicarboxylato)ruthenium(II) (N719) were purchased from OPV Tech Co., Ltd. These chemicals were used in their as-received state without any additional purification steps.

### 2.2. Synthesis of Cu_2_FeSnS_4_ Nanosheets

The experiments were conducted within a fume hood using standard Schlenk techniques under an inert N_2_ atmosphere. For the synthesis of CFTS nanosheets, copper (II) acetylacetonate (0.5236 g, 2 mmol), ferric chloride hexahydrate (0.2703 g, 1 mmol), and tin (IV) chloride pentahydrate (0.3506 g, 1 mmol) were combined in a 100 mL four-neck round-bottom flask with 15 mL of 1-dodecanethiol. The mixture underwent gradual heating to 120 °C, followed by maintenance at this temperature for 30 min to completely remove residual moisture from the system. Then, the mixture was heated until reaching 200 °C, resulting in a brown coloration. Subsequently, the mixture was heated to 260 °C at a rate of 2 °C/min and stirred continuously for 60 min at this temperature. After cooling the mixture to room temperature, the resulting black precipitate was washed three times with a mixture of hexane and ethanol (1:3) by centrifugation (9000 rpm/min). In this process, the residual organic groups were removed to improve the dispersibility of materials. Finally, the power was dried to obtain CFTS nanosheets. The other samples in this work were prepared under the same experimental conditions as above by changing the corresponding heating time and temperature.

### 2.3. Ligand Exchange with S^2−^

In order to substitute the native ligands with S^2−^ anions, as-synthesized CFTS nanosheets dispersed in hexane (20 mg/mL) were combined with a solution containing (NH_4_)_2_S (2 mL of ammonium sulfide in 20 mL of formamide), followed by stirring the mixture for 2 h. Subsequently, the mixture was allowed to undergo phase separation. The CFTS nanosheets were then transferred to the formamide phase, resulting in a clear hexane layer, which indicated a successful ligand exchange. The hexane layer was carefully removed, and fresh hexane was introduced to the formamide phase. The mixture was vigorously shaken to eliminate any remaining organic ligands from the formamide phase.

### 2.4. Fabrication of DSSCs

The CFTS thin films were fabricated using a spray-painting technique. Initially, the ligand-exchanged CFTS nanosheets were dispersed in ethanol to create an ink with a concentration of 20 mg/mL. The suspension was then spray-deposited onto FTO glass, and the resulting films were annealed at 300 °C for 30 min under an Ar atmosphere. A Pt electrode (~5 nm) was deposited onto the FTO glass via direct current sputtering in an Ar atmosphere (deposition pressure: 10^−2^ Pa, current 15 mA). Before use, the TiO_2_ photoanodes underwent treatment at 100 °C for 30 min. To prepare the dye solution, a quantity of 0.3 mM N719 dye was solubilized in absolute ethyl alcohol. The dye absorption onto the anode was achieved by immersing the electrode in the dye solution at 30 °C overnight. Finally, DSSCs were fabricated using the TiO_2_ photoanodes and CFTS/Pt CEs, which were sealed using a Polytetrafluoroethylene (PTFE) tape film (thickness: ~50 μm).

### 2.5. Characterizations

The nanosheets’ crystal structure underwent characterization using an X-ray diffractometer (XRD, Rigaku-Smart Lab Advance, Rigaku, Tokyo, Japan). Field-emission scanning electron microscopy (FESEM, HITACHI S−4800, HITACHI, Tokyo, Japan) was employed to analyze the samples’ morphology and microstructure. For investigating crystallinity and microstructure details, high-resolution transmission electron microscopy (TEM, JEOL JEM−2100, JEOL, Tokyo, Japan) was conducted. The Raman test was conducted through a Horiba Labram HR 800 spectrometer with a 15 mW green argon laser (λ = 532 nm) serving as the excitation light source. To evaluate DSSC performance, photocurrent density-voltage (J−V) curves were measured under AM1.5 simulated solar light with a black background.

## 3. Results

### 3.1. Structure Characterization of CFTS Nanosheets

**Figure 1:**
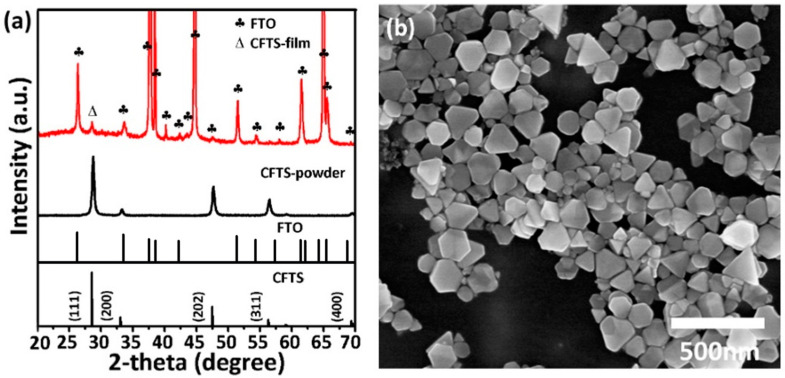
(**a**) XRD patterns of as-prepared CFTS nanosheets powders and prepared CFTS film. (**b**) FESEM image of CFTS nanosheets. Standard XRD patterns for CFTS (JCPDS # 70-4373) and FTO (JCPDS #73-1667) are also provided.

The crystal structure and morphology of the CFTS nanosheets were examined using XRD and TEM techniques, as illustrated in Figure 1. The XRD pattern (Figure 1a) of the synthesized CFTS nanosheets reveals diffraction peaks corresponding to the (111), (200), (202), (311), and (400) planes of cubic zinc blende phase CFTS. These peaks match well with the standard JCPDS pattern (no. 70-4373). Additionally, the XRD pattern of the sprayed CFTS films displays peaks originating from the FTO substrate and a prominent peak at 28.5° corresponding to the (111) plane of CFTS. Considering the 2D morphology of CFTS, we can speculate that sprayed CFTS thin films tend to have an axial orientation, resulting in a highly exposed (111) crystal plane. Raman spectroscopy was employed to verify the phase purity of CFTS, as Cu–Sn–S compounds often present overlapping diffraction peaks. The observed Raman peak at 318 cm^−1^ aligns (Figure S1, Supporting Information) in accordance with the reported value and can be attributed to symmetric vibrational motion of the sulfur atom, which indicates the successful synthesis of CFTS [41,42]. Figure 1b displays synthesized CFTS nanosheets exhibiting a nearly hexagonal and triangular morphology, with lateral sizes ranging from 30 to 300 nm and thicknesses of 25 to 35 nm.

**Figure 2:**
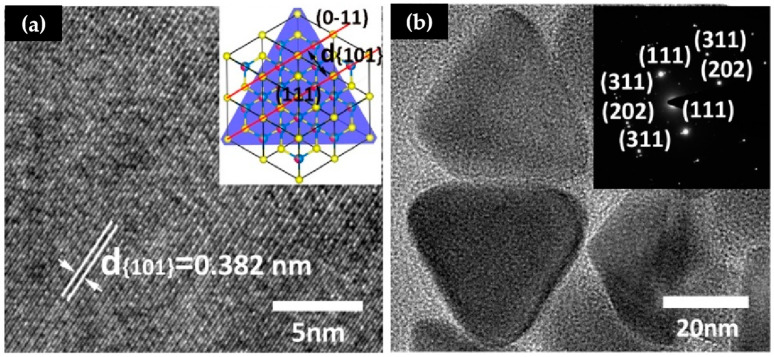
(**a**) HRTEM image of CFTS nanosheets and illustration of its crystal structure. (**b**) TEM image of CFTS nanosheets and SAED pattern.

Examining the HRTEM image (Figure 2a) reveals lattice fringes with a d-space of 0.382 nm, corresponding to the (101) planes of zinc blende CFTS. The SAED pattern, shown as an inset in Figure 2b, confirms a well-ordered structure and exhibits a cubic close packing arrangement of face-centered cubic 2D CFTS nanosheets. The SAED pattern’s highly periodic spots can be indexed to the (111), (311), and (202) planes of standard bulk CFTS (JCPDS #70-4373). Further investigation of the CFTS nanosheets’ composition was conducted using EDS, which indicated a Cu/Fe/Sn/S concentration close to the expected 2:1:1:4 ratio (Figure S2 and Table S1, Supporting Information).

**Figure 3:**
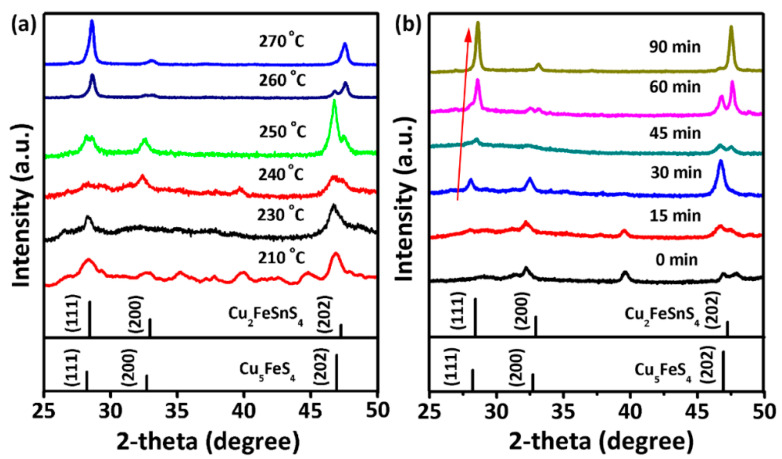
XRD patterns of nanocrystals synthesized (**a**) at gradually increased reaction temperatures from 210 to 270 °C, and (**b**) with various reaction times from 0 to 90 min at 240 °C. Standard XRD patterns for Cu_2_FeSnS_4_ (JCPDS # 70-4373) and Cu_5_FeS_4_ (JCPDS #73-1667) are also provided.

**Figure 4:**
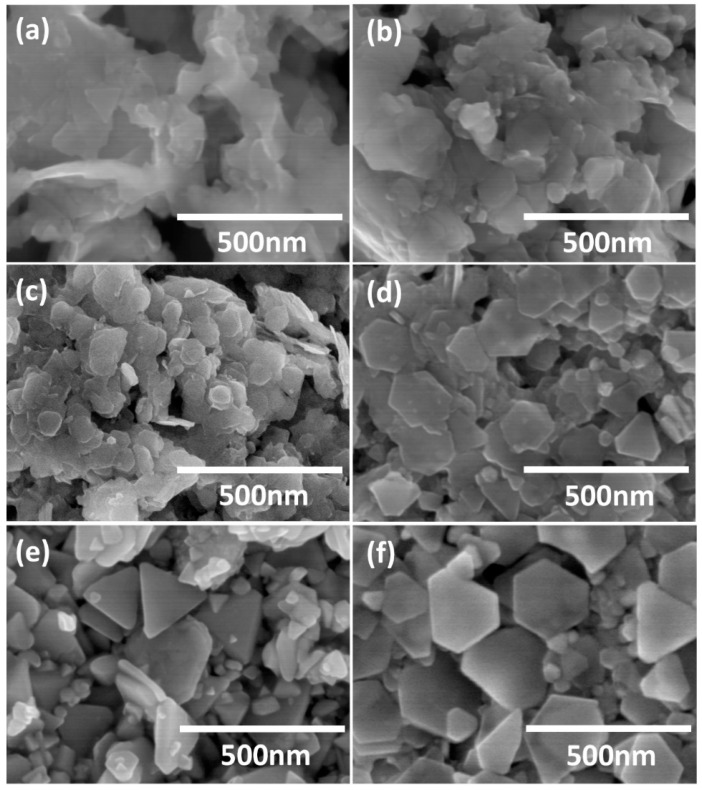
SEM images of nanocrystals synthesized by the reaction of Cu(acac)_2_, FeCl_3_·6H_2_O, SnCl_4_·5H_2_O, and 1-DDT at (**a**) 210 °C, (**b**) 230 °C, (**c**) 240 °C, (**d**) 250 °C, (**e**) 260 °C, and (**f**) 270 °C.

**Figure 5:**
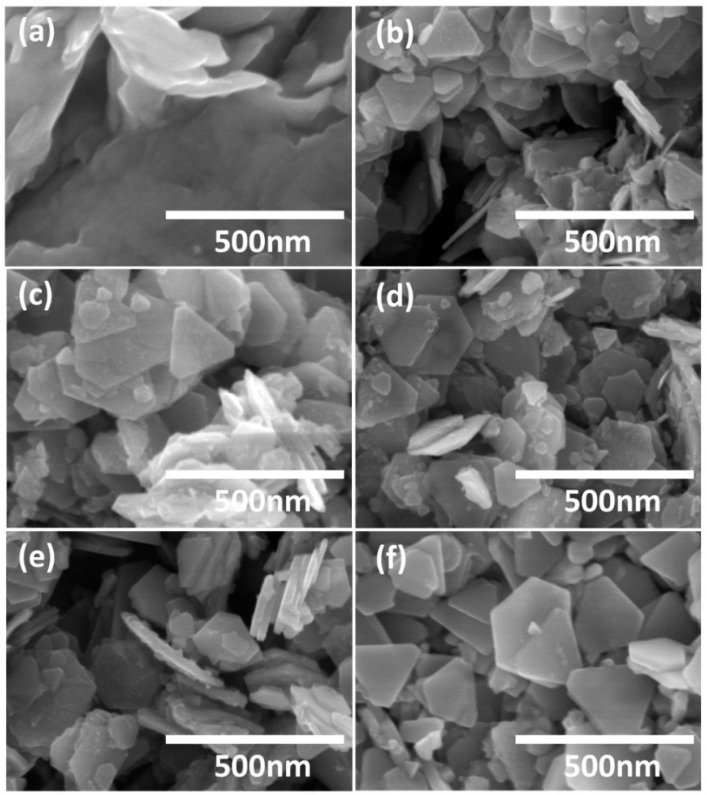
SEM images of nanocrystals synthesized by the reaction of Cu(acac)_2_, FeCl_3_·6H_2_O, SnCl_4_·5H_2_O, and 1-DDT for (**a**) 1 min, (**b**) 15 min, (**c**) 30 min, (**d**) 45 min, (**e**) 60 min, and (**f**) 90 min.

To reveal the formation mechanism of CFTS nanosheets, we systematically investigated the impact of reaction temperature and time on the crystal structure of the prepared nanocrystals. The phase purity, crystallinity, and morphology of the obtained nanocrystals were determined through XRD, EDS, and SEM measurements. Figure 3 presents the XRD patterns of the CFTS nanosheets, intermediate products, and the standard diffraction patterns of bulk CFTS and Cu_5_FeS_4_. Below 250 °C (Figure 3a), the formation of Cu_5_FeS_4_ nanocrystals is thermodynamically favored. At a reaction temperature of 260 °C, the XRD pattern demonstrates a higher intensity of CFTS compared to Cu_5_FeS_4_, indicating the prevalence of CFTS in the product. Further increasing the reaction time, phase-pure CFTS can be achieved at 60 min (Figure S3). Additionally, a series of experiments were conducted with various reaction times ranging from 1 to 90 min, maintaining a fixed reaction temperature of 240 °C. The XRD patterns of the resulting products are shown in Figure 3b. We notice that Cu_5_FeS_4_ is formed at the very beginning until 30 min, but the intensity of CFTS gradually increases with longer reaction times up to 90 min. Products obtained at the reaction time of 30 min can be well indexed to bornite Cu_5_FeS_4_ (JCPDS No. 73-1667), and its composition has also been confirmed using EDS, which is very close to the 5:1:4 ratio (Table S1, Supporting Information). A systematic right-shift in the diffraction peak positions is observed with increasing reaction time, indicating lattice compression due to the formation of CFTS. A steady enhancement of the (111) peak intensity is also noted with increasing reaction time. With reaction times longer than 90 min, phase-pure CFTS can be obtained with complete phase transformation of the Cu_5_FeS_4_. The diffraction peaks for all the patterns can be indexed to Cu_5_FeS_4_ and CFTS, with an absence of any binary and ternary sulfide impurity phases. The shape and size of products obtained from temperature-dependent and time-dependent one-pot heating experiments have been measured by FESEM, as shown in Figure 4 and Figure 5. All the temperature-dependent and time-dependent reactions result in the formation of nanosheets with nearly hexagonal and triangular shape. The size and thickness of obtained nanosheets increase with longer reaction times and higher reaction temperatures.

**Figure 6:**
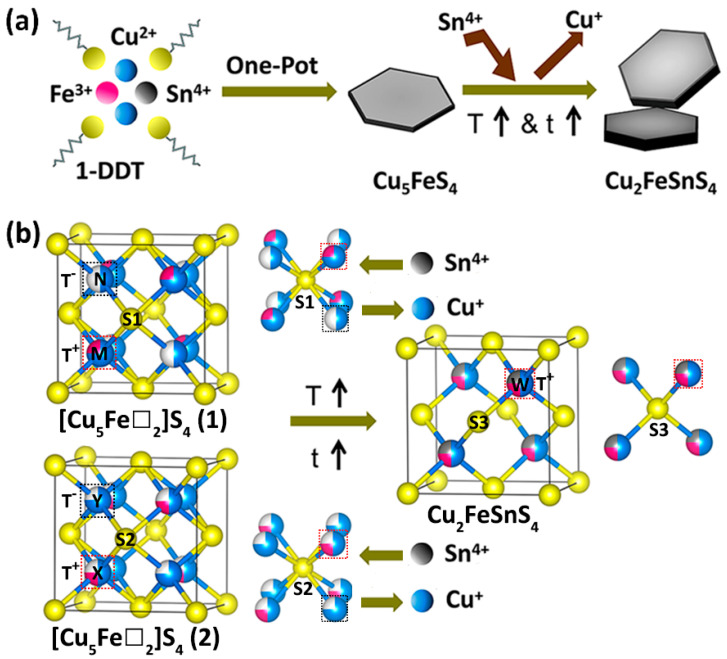
(**a**) Schematic illustration of synthesis process of CFTS nanosheets. (**b**) Schematic illustration of the conversion mechanism of Cu_5_FeS_4_ to CFTS. [Cu_5_Fe□_2_]S_4_: statistical distribution of copper (blue), iron (red), and vacancies (white); Cu_2_FeSnS_4_: statistical distribution of copper (blue), iron (red), tin (gray), and vacancies (white).

Based on the aforementioned findings, we examined the process conditions involved in synthesizing CFTS nanosheets. In light of these outcomes, we put forth a plausible growth mechanism, as depicted in Figure 6a. We found that it is inevitable to form Cu_5_FeS_4_ nanosheets at lower temperatures during the heating process. To validate the above statement, we used the same reactants to conduct a one-pot heating process with a reaction temperature of 210 °C for different reaction times from 0 to 60 min. The results show that low temperature is ineffective for the synthesis of CFTS nanosheets, as confirmed by the XRD patterns shown in Figure S4. The obtained phase pure Cu_5_FeS_4_ nanosheets show a thinner hexagonal and triangular morphology (Figure S5, Supporting Information). With an increase in temperature and time, the gradual reaction of SnCl_4_·5H_2_O occurs with Cu_5_FeS_4_ nanosheets and 1-DDT, leading to the formation of CFTS nanosheets. This conversion process involves the diffusion of Sn^4+^ ions into the Cu_5_FeS_4_ nanosheets, enhancing the movement and rearrangement of cations within the Cu_5_FeS_4_ lattice. As the Sn concentration increases, the smaller Sn ion radius (Sn^4+^, 55 pm at CN = 4; Cu^+^, 60 pm at CN = 4) results in a noticeable lattice compression. Consequently, there is an observable shift in the (111) diffraction peak positions toward higher angles, as shown in Figure 3.

Due to the complication of the significant differences between Cu(I) and Sn(IV) electron configurations, the CFTS nanosheet formation mechanism involves not only the simple replacement of Sn for Cu in Cu_5_FeS_4_. Moreover, bornite Cu_5_FeS_4_ has been reported to exhibit three structural forms depending on the temperature of both synthetic and natural bornites [43]. The ordered orthorhombic structure (Pbca, *a* = 10.95 Å, *b* = 21.86 Å, *c* = 10.95 Å) is the most stable crystal structure at low temperatures. With increasing temperature, it will transfer to an intermediate cubic semi-ordered structural structure (Fm3$\bar{m}$, a = 10.98 Å). Above 240 °C, a high temperature disordered cubic structural structure (Fm3$\bar{m}$, a = 5.50 Å) will dominate at the expense of an intermediate cubic semi-ordered structural structure. Above all, we propose two possible high-temperature cubic structures for intermediate Cu_5_FeS_4_ nanosheets, as shown in Figure 6b. The metal-deficient anti-fluorite structure of both high-temperature cubic forms is characterized by a random distribution of iron, copper, and cationic vacancies within the tetrahedral sites of the cubic face-centered sulfide ion framework. This arrangement is described by the formula [Cu_5_Fe□_2_]S_4_, where □ represents the vacancy. The two proposed basic unit sub-cells present highly random metal atom distribution: in the first unit sub-cell, [Cu_5_Fe□_2_]S_4_ (1), half of the tetrahedral voids (T^+^) are occupied by composite atoms M (75 at% Cu^+^ 25 at% Fe) and the rest of the tetrahedral voids (T^−^) are occupied by composition atoms N (50 at% Cu^+^ 50 at% vacancy), and the XRD pattern based on this structure is showed as the Simulated 1 in Figure S6. Meanwhile, in the second unit sub-cell, [Cu_5_Fe□_2_]S_4_ (2), the tetrahedral voids are occupied half and half by the composite atoms X [50 at% Cu^+^ 25 at% Fe^+^ 25 at% vacancy] in T^+^ and composite atoms Y (75 at% Cu^+^ 25 at% vacancy) in T^−^, respectively. The XRD pattern based on this structure is shown as the Simulated 2 in Figure S6. Drawing upon the aforementioned considerations, we put forth a potential mechanism for the transformation process from the Cu_5_FeS_4_ structure to the CFTS structure. α is the valence electrons of the cations surrounding an anion; β is the anion’s coordination, and γ is the number of valence electrons of the anion. If ∑ = α/β + γ = 8, the structure will adopt a low-energy state because of the closed-shell configuration of the anion, ensuring its stability. In the disordered [Cu_5_Fe□_2_]S_4_ (1, 2) cubic structures, the S^1−^[5 Cu(I), Fe(III)] and S^2−^[5 Cu(I), Fe(III)] clusters, both with ∑ values equal to 7, are both metastable during the one-pot heating process. When increasing the temperature, the Sn(IV) ion, which favors tetrahedral coordination with the lowest-lying full-filled 4d orbital, one must substitute 25% Cu atoms in M located at T^+^ of [Cu_5_Fe□_2_]S_4_ (1) or fill vacancies in X located at T^+^ of [Cu_5_Fe□_2_]S_4_ (2), resulting in S1 and S2 tetrahedra surrounded by two Cu atoms, one Fe atom, and one Sn atom. Meanwhile, three Cu atoms of each S^1−^[5 Cu(I), Fe(III)] and S^2−^[5 Cu(I), Fe(III)] cluster are “driven out” with each T^−^ being vacant in order to minimize the internal energy. Moreover, this disturbs the local neutrality of the left half-filled tetrahedral voids (T^+^). So, the Fe(III) ion in this disordered structure with a half-filled 3d^5^ orbital transfer to a more stable electron configuration of 3d^5^4s^1^, with both 3d^5^ and 4s orbitals half-filled based on Hund’s rule. Figure 6b illustrates the unit cell of zinc blende CFTS, where the arrangement can be visualized as a face-centered cubic lattice of S^2−^ anions. Within this lattice, half of the available tetrahedral voids filled with W (50 at% Cu^+^ 25 at% Fe^+^ 25 at% Sn) cations, preferentially form S^3−^[2 Cu(I), Fe(II), Sn(IV)] clusters to reach ∑ = 8 and lower the internal energy with charges in equilibrium, which is inconsistent with the crystal parameters of stimulated XRD data. Ongoing experiments are being conducted to gain a comprehensive understanding of the intricate mechanism underlying the conversion. Throughout the transformation from Cu_5_FeS_4_ nanosheets to CFTS nanosheets, the entire process, as described earlier, occurs in conjunction with the adoption of a disordered lowest-energy structure.

### 3.2. Photovoltaic Measurements

**Figure 7:**
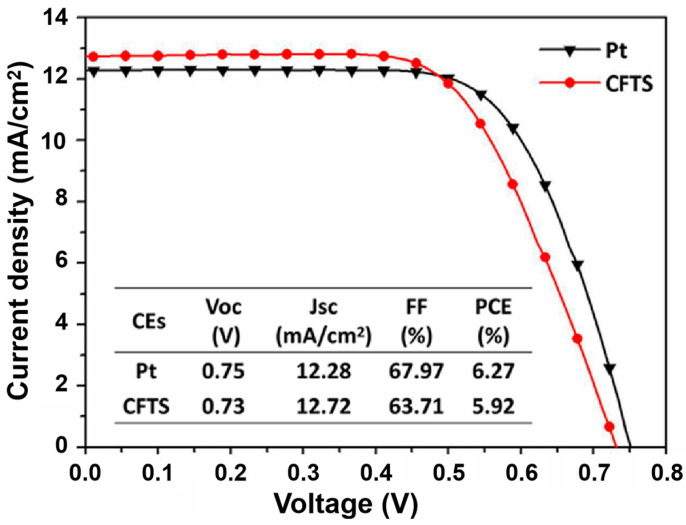
Current density (J) versus voltage (V) plots of DSSCs fabricated using CFTS nanosheets and Pt.

To assess the viability of CFTS as a potential substitute for Pt in DSSCs, DSSCs were created utilizing CFTS thin films as the counter electrode. It is worth pointing out that, during the assembly and testing process of DSSCs, the humidity or oxygen levels of environments were maintained at normal levels, suggesting the excellent tolerance and stability of CFTS thin film and DSSCs. The investigation included examining the J−V curves of the DSSCs fabricated with CFTS nanosheets and Pt (counter electrode), as well as presenting a comprehensive summary of the photovoltaic parameters of the DSSCs. These parameters encompass the open-circuit voltage (V_OC_), short-circuit current density (J_SC_), fill factor (FF), and power conversion efficiency (P_CE_), as depicted in Figure 7. DSSCs fabricated using CFTS and annealed at argon atmosphere without any selenization and sulfuration had a 5.92% PCE with J_SC_ = 12.72 mA/cm^2^, V_OC_ = 0.73 V, and FF = 63.71%. This power conversion efficiency is competitive compared to CFTS/CCTS/CNTS (2.73%), Cu_2_FeSnS_4_ powder (1.32%), and p-type Cu_2_FeSnS (2.9%) [26,44,45]. DSSCs fabricated using Pt had a 6.27% PCE with J_SC_ = 12.28 mA/cm^2^, V_OC_ = 0.75 V, and FF = 67.97%. It is evident that CFTS nanosheets exhibit a similar power conversion efficiency with a Pt counter electrode based on the DSSC performance.

## 4. Conclusions

In conclusion, we have successfully devised a straightforward and highly effective one-pot synthesis approach for producing 2D CFTS nanosheets. The characterization results of SEM, XRD, et al. indicate that CFTS possesses a 2D hexagonal plate-like structure with exposed high-energy (111) crystal facets. By varying the synthesis temperature and time of CFTS, we propose a possible mechanism for the transformation of Cu_5_FeS_4_ into CFTS. Based on the octahedral rule, we propose that the [Cu_5_Fe□_2_]S_4_ (1, 2) of the cubic structural phase remains in a metastable state at high temperatures. Therefore, Sn(IV) tends to diffuse into the Cu_5_FeS_4_ lattice to reduce internal energy and form CFTS. When utilized as a counter electrode in DSSCs, the CFTS thin film demonstrates a favorable power conversion efficiency of 5.92%. Furthermore, we believe that based on further doping modification, annealing or assembly with other materials, such as graphene, can further enhance its performance as an electrode for DSSCs. The outcome of our work indicates that CFTS holds substantial promise as an economical substitute for Pt in DSSCs, providing a cost-effective alternative. 

## Data Availability

The data that support the findings of this study are available from the corresponding author upon reasonable request.

## Supplementary Materials

The following supporting information can be downloaded at: https://www.mdpi.com/article/10.3390/ma16134743/s1.
