# Supplementary material for: Synthesis of Single Crystal 2D Cu2FeSnS4 Nanosheets with High-Energy Facets (111) as a Pt-Free Counter Electrode for Dye-Sensitized Solar Cells
Source: Materials (Basel). 2023 Jun 30;16(13):4743. doi: 10.3390/ma16134743 (PMC10342958; doi:10.3390/ma16134743)
Supplement: Supplementary file 1 [file materials-16-04743-s001.zip › materials-2465799-SI.pdf]

Supporting Information

Synthesis of single crystal 2D Cu_2FeSnS_4 nanosheets with high-energy facets (111) as a Pt-free counter electrode for dye-sensitized solar cells

Jianming Wen, Suqin Chen, You Xu, Tuxiang Guan, Xiaoyan Zhang*, and Ningzhong Bao*

State Key Laboratory of Materials-Oriented Chemical Engineering, College of Chemical Engineering, Nanjing Tech University, Nanjing, Jiangsu 210009, P. R. China.

Content

Table S1--------------------------S2
Figure S1-----------------------S2
Figure S2-----------------------S3
Figure S3-----------------------S3
Figure S4-----------------------S4
Figure S5-----------------------S4
Figure S6-----------------------S5

Table. S1 Elemental composition of synthesized nanosheets

Atoms / Conditions	Atomic percentage (%)			
	**Cu**	**Fe**	**Sn**	**S**
**240°C, 30 min**	48.15	10.27	0	41.57
**260°C, 60 min**	24.53	13.84	12.75	48.88

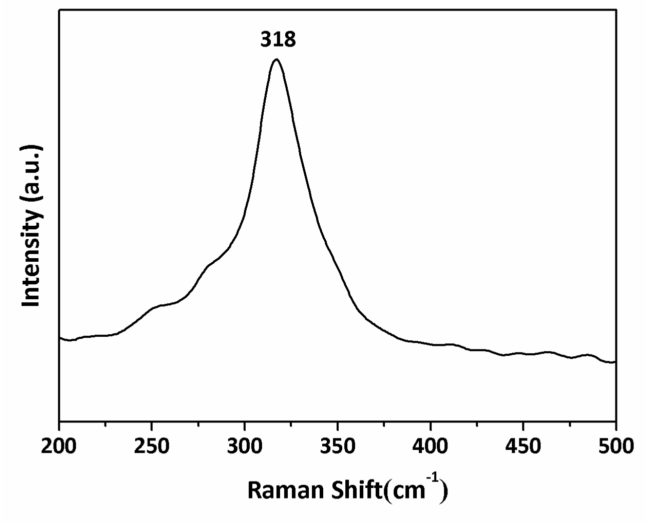

Figure. S1 Raman spectrum of synthesized CFTS nanosheets.

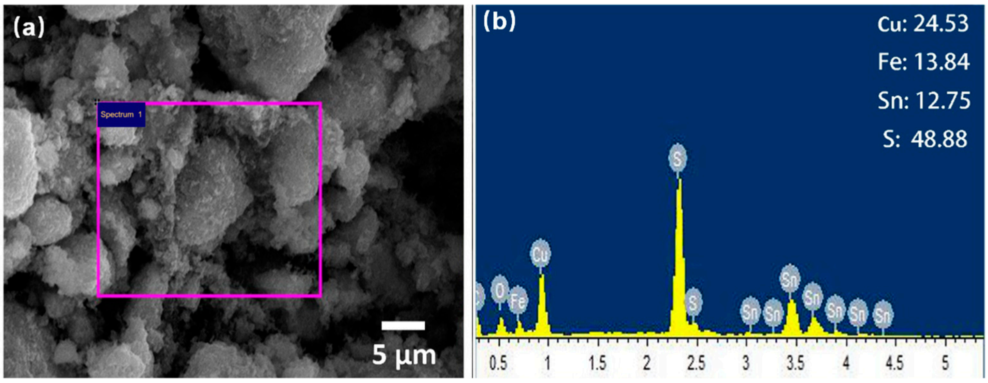

Figure S2 EDS spectrum and elemental composition of synthesized CFTS nanosheets. (a) Dark-field TEM image of CFTS, (b) atomic ratio of CFTS

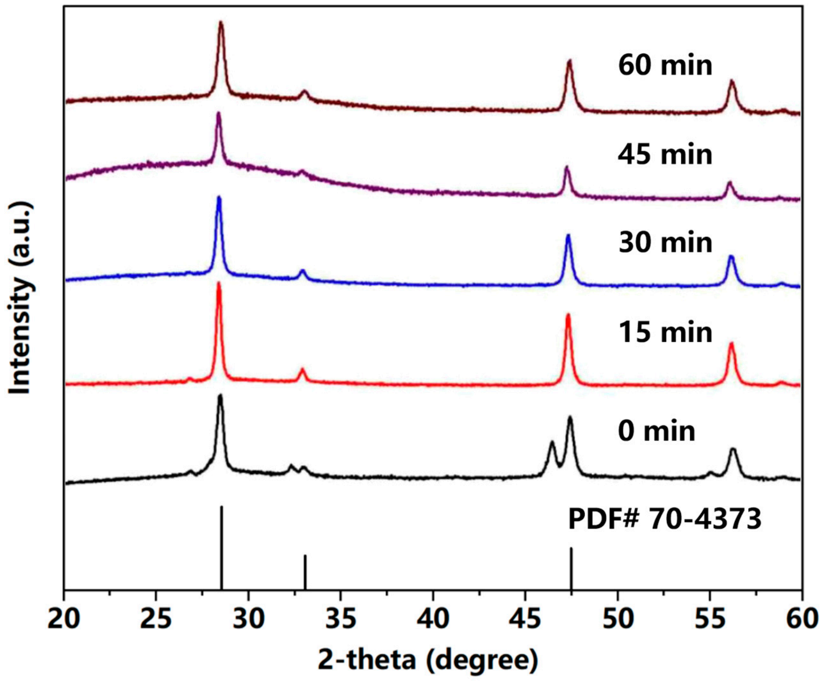

Figure. S3 XRD patterns of nanocrystals synthesized with various reaction times from 0 to 60 min at 260 °C. Standard XRD patterns for Cu_2FeSnS_4 (JCPDS # 70-4373) is also provided.

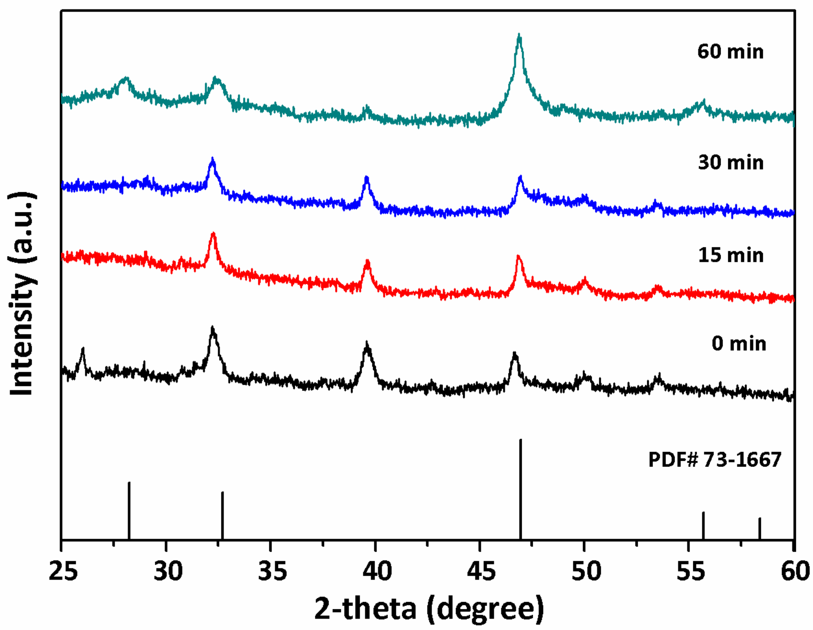

Figure. S4 XRD patterns of as-synthesized nanocrystals prepared by the reaction of $Cu(acac)_2$, $FeCl_3·6H_2O$, $SnCl_4·5H_2O$, and 1-DDT with different reaction times from 0 to 60 min at 210 °C.

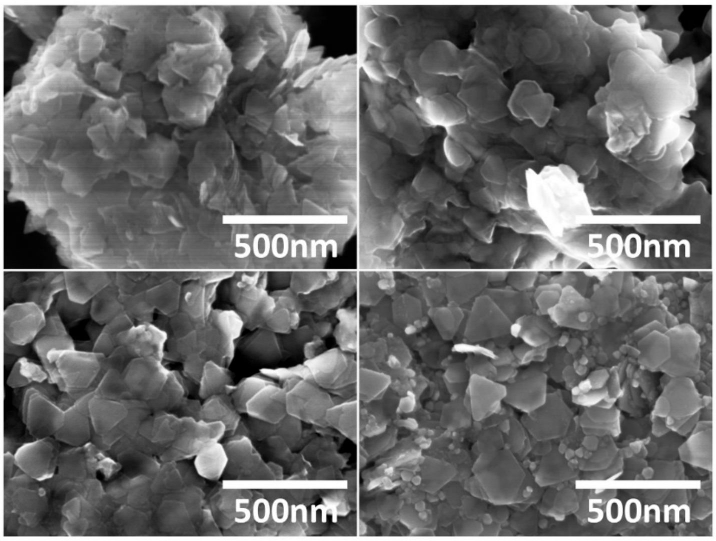

Figure. S5 SEM images of as-synthesized nanocrystals prepared by the reaction of $Cu(acac)_2$, $FeCl_3·6H_2O$, $SnCl_4·5H_2O$, and 1-DDT with different reaction times from 0 to 60 min at 210 °C.

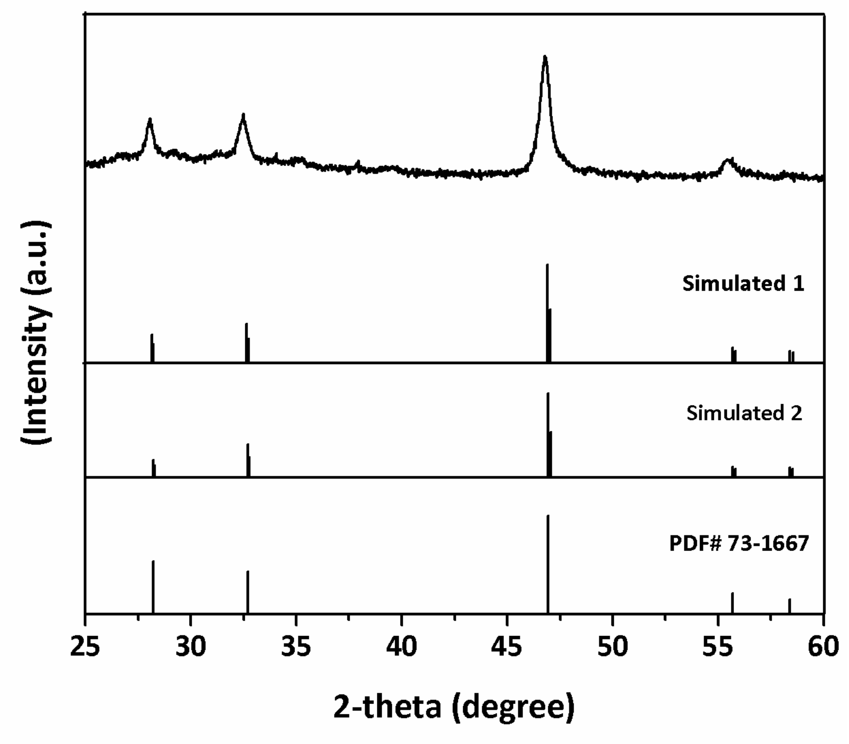

Figure. S6 The XRD pattern of Cu_5FeS_4.
